# Persistence of Anti-Hbs after up to 30 Years in Health Care Workers Vaccinated against Hepatitis B Virus

**DOI:** 10.3390/vaccines9040323

**Published:** 2021-04-01

**Authors:** Silvia Cocchio, Vincenzo Baldo, Anna Volpin, Marco Fonzo, Annarosa Floreani, Patrizia Furlan, Paola Mason, Andrea Trevisan, Maria Luisa Scapellato

**Affiliations:** 1 Department of Cardiac Thoracic and Vascular Sciences, and Public Health, University of Padua, 35100 Padova, Italy; silvia.cocchio@unipd.it (S.C.); anna.volpin@aopd.veneto.it (A.V.); marco.fonzo@unipd.it (M.F.); patrizia.furlan@unipd.it (P.F.); paola.mason.1@unipd.it (P.M.); andrea.trevisan@unipd.it (A.T.); marialuisa.scapellato@unipd.it (M.L.S.); 2 Scientific Institute for Research, Hospitalization and Healthcare Negrar, 37024 Negrar, Italy; annarosa.floreani@unipd.it; 3 Department of Surgical, Oncological and Gastroenterological Sciences, University of Padua, 35124 Padova, Italy

**Keywords:** HBV, vaccination, anti-HBs, health care workers

## Abstract

The burden of hepatitis B virus (HBV) infection is a serious public health problem all over the world. Vaccination remains the most effective prevention measure, and safe and effective HBV vaccines have been available since 1982. Health care workers (HCWs) vaccinated against HBV and prospectively followed up for at least 14 years were classified by their antibody titers after primary vaccination as: poor responders (10–99 mIU/mL); moderate responders (100–999 mIU/mL); and good responders (≥1000 mIU/mL). The incidence of antibody loss was calculated for 1000 person-years and the anti-HBs persistence was calculated. The analysis concerned 539 HCWs: 494 good responders (91.7%); 37 moderate responders (6.9%); and eight poor responders (1.5%). The incidence of anti-HBs loss was 52.1 per 1000 person-years for the poor responders, 11.3 per 1000 person-years for the moderate responders, and 1.4 per 1000 person-years for the good responders. The mean persistence of anti-HBs differed significantly between the three groups, being: 19.2 years (95% CI: 15.6–22.8), 25.4 years (95% CI: 23.0–27.9), and 31.0 years (95% CI: 30.5–31.5) for the poor, moderate and good responders, respectively. In conclusion, our findings demonstrate a good persistence of protective anti-HBs titers in HCWs exposed to occupational risk for up to 30 years after a primary vaccination cycle (even without a booster dose) if their titer was initially higher than 100 mIU/mL.

## 1. Introduction

Hepatitis B virus (HBV) infection remains a public health problem all over the world. Around 250 million people live with chronic HBV infection [1,2] and roughly 30% of the world’s population would show serological evidence of current or past infection [3]. Most acute infections are asymptomatic. Over the years after contracting the virus, approximately 15–40% of chronically-infected patients gradually develop serious HBV-related symptoms. These symptoms of their hepatitis may involve cirrhosis, liver failure, and hepatocellular carcinoma, and nearly a million people around the world die every year due to the complications of HB-related disease [4].

Although HBV infection is a globally relevant concern, not all regions in the world are equally affected and the prevalence of hepatitis due to the HBV shows significant variations across countries: the prevalence is the lowest in Northern America and Western Europe, where less than 2% of the general population is estimated to be positive to the HBaAg; it ranges between 2% and 8% in the Mediterranean Region, Eastern Europe, and most Asian countries; it stands above 8% in some low income (LIC) and low and middle-income countries (LMIC) in Asia and Sub-Saharan African countries [5].

In 2017, 30 EU/EEA Member States reported 26,907 cases of HBV infection. Excluding the five countries that only reported acute cases, the number of cases identified (26,262) amounts to a crude rate of 6.7 per 100,000 population. The number of acute cases seen in Europe continues to decline, consistently with global trends, and most likely reflects the impact of national vaccination programs [6]. In this regard, Italy is not an exception, and the epidemiology of HBV infection showed a noteworthy change during the last five decades. A substantial and progressive reduction has been seen in both the incidence of acute hepatitis B cases (dropping from 10 cases per 100,000 population in 1984 to less than 1 case—0.85, to be precise—per 100,000 population in 2012) and the prevalence of HBsAg chronic carriers (moving from 3% in the early 1980s to less than 1% in 2010). The reasons for this achievement are health-related but also social: an improvement in the socioeconomic level of the general population; a reduction in the number of large families in which HBV transmission often occurs between siblings; the effects of educational campaigns aimed primarily at containing the spread of the HIV infection; the availability of HBV screening test for pregnant women and, last but not least, the implementation of a nationwide universal vaccination program [5].

Compared with the general population, healthcare workers (HCWs) are at higher risk for transmission of a wide range of blood-borne infectious agents, including—by way of example but not limited to—the hepatitis B virus (HBV), the hepatitis C virus (HCV) and the human immunodeficiency virus (HIV). Of the above pathogens, the HBV is far more infectious than both HCV and, even to a greater extent, HIV [7]. HCWs are more exposed to the risk of acquiring HBV infection than members of the general population due to the frequent exposure of their eyes, oral mucosa, and skin to patients’ potentially infectious blood. They may also contract the disease percutaneously from contaminated sharp objects (needles, blades, etc.) [8,9]. The annual estimated proportion of HCWs exposed to HBV is 5.9%, which corresponds to an estimated 66,000 preventable HBV infections each year among HCWs worldwide [10].

The adoption of numerous different measures and interventions, and the use of standard precautions and safety devices have helped to reduce the risk of HBV transmission, but the development of vaccines against HBV has undeniably been one of the most important achievements in terms of the prevention of HBV infection [7].

HCWs represent one of the most important groups to protect against HBV infection in adults [11,12]. Safe and effective HBV vaccines have been commercially available since 1982. After the first plasma-derived vaccines, recombinant DNA vaccines were licensed. Yeast-product vaccines (the most widely used) are manufactured by inducing the expression of HBsAg protein in genetically-engineered yeast cells that contain the S gene. The protective efficacy of HBV vaccination stems from the induction of anti-HBs antibodies at concentrations above 10 mIU/mL [13]. It has been demonstrated that these vaccines are safe when administered to infants, children, adolescents, and adults. The side-effects most frequently reported in people receiving hepatitis B vaccine are pain at the injection site (in 3% to 29% of cases) and a transient mild fever >99.9 °F (>37.7 °C) (in 1% to 6% of individuals) [14].

Studies indicate that immunological memory of HBV remains intact for at least 30 years [15,16]. Immunocompetent individuals who achieve hepatitis B surface antibody (anti-HBs) concentrations of ≥10 mIU/mL after being vaccinated prior to any exposure to the virus are protected against both acute disease and chronic infection [17]. This remains true even if their anti-HBs levels, once measured and found adequate, subsequently drop even to below detectable levels. If exposed to HBV, people whose immune systems are competent will mount an anamnestic response and develop protective anti-HBs [16]. Smoking, obesity, genetic factors, male gender, pre-existing rheumatic disease, and immune suppression are associated with a diminished immune response to hepatitis B vaccination, although not all evidence is unanimous regarding the factors listed above [18,19,20]. The effect of obesity or obesity-related diseases on HBV vaccine-escape mutation remains unclear, but the impaired vaccine response in obesity still poses a challenge in reaching effective immunization against HBV [21]. In addition, although immunocompromised individuals have lower immunogenicity, those who achieve and maintain a protective antibody response before exposure to HBV nonetheless have a high level of protection against infection [22].

It has generally been reported that an individual’s antibody titers decline over the years after their immunization. This coincides with a gradually increasing rate of infection, especially when the titer of the antibody against hepatitis B surface antigen (HBsAg) falls below the protective cut-off level of 10 mIU/mL [23]. However, variation in policies on booster dose administration for HCWs in Europe and the USA has been reported, with wide differences between no recommendation for adults whose immune status is normal in the USA, and recommendation for booster dose within one year in case of anti-HBs titer <100 mIU in Germany. A recent statement from the Consensus Committee Group of Experts does not recommend a booster dose for HCWs but recommends immunization for any health care workers for whom a vaccination record cannot be traced. Moreover, they recommend post-vaccination antibody testing and administration of an additional dose of vaccine in cases of inadequate immunological priming [24]. Studies are ongoing to ascertain whether booster doses of hepatitis B vaccine will be needed in the future [16].

We started a vaccination campaign for groups at risk of HBV in 1983. We subsequently studied the persistence of seroprotection induced by HBV vaccination in 422 HCWs from 4.8 to 18.8 years after their primary immunization, and 107 of these HCWs had received a booster dose 6 years after primary immunization with plasma- or yeast-derived vaccines [25]. In the cohort not given a booster dose, it emerged that the cumulative proportion of HCWs still protected against HBV after 10 years was >85%. The study thus concluded that a booster dose is not necessary for healthy adults, since more than 80% of responders to vaccination still had protective antibody titers detectable after 10 years, and bearing in mind that immunological memory persists for more than 10 years. Our prospective study on the effects of offering vaccination to HCWs has continued over time and, within this context, we have also looked at the cohort of individuals not given a booster dose. Following them up to assess the persistence of anti-HBs can be useful for the purpose of public health considerations on the best immunization strategies for HCWs.

## 2. Material and Methods

### 2.1. Context

Our vaccination campaign started in January 1983 for all individuals belonging to groups at high risk of becoming infected with the HBV virus. HCWs found negative for HBV markers (HBsAg, anti-HBs, and anti-HBc) were offered vaccination against HBV with:

(1) plasma-derived vaccines (HEVAC-B (Pasteur, Merieux, Lyon, France) at 0, 1, 2, and 14 months or HB-Vax (Merck Sharp and Dohme, Sumneytown Pike, Westpoint, PA, USA) at 0, 1, and 6 months), up until the end of 1989;
(2) yeast-derived vaccines (Engerix-B (Smith Kline Beecham Biological, Rixensart, Belgium) and Recombivax (Merck Sharp and Dohme)) at 0, 1, and 6 months, as of 1990.

### 2.2. Study Population

HCWs vaccinated during the campaign against HBV were prospectively followed up for at least 14 years. Their anti-HBs antibody titers were measured at the end of the primary immunization cycle, and then at various different times afterward, as part of the HCWs’ preventive medical check-ups according to the Occupational Health Protocol. The anti-HBs antibody titers of the end of the primary vaccination cycle and as at the latest available measurement (no later than February 2019) were considered in our study on a cohort of HCWs not given a booster dose.

### 2.3. Laboratory Methods

HBV markers (HBsAg, anti-HBs, and anti-HBc) were tested, always at the same laboratory, by radioimmunoassay (AUSIA II, AUSAB, and CORAB B, Abbott Labs, Chicago, IL, USA) according to the manufacturer’s recommendations. As generally accepted, vaccinated subjects were considered as seroprotected when their anti-HBs titers were ≥10 mIU/mL. After primary vaccination, the HCWs were classified by antibody titer as: (i) poor responders (10–99 mIU/mL); (ii) moderate responders (100–999 mIU/mL); and (iii) good responders (≥1000 mIU/mL). Antibody loss and antibody persistence were defined as testing negative or positive for anti-HBs, respectively, according to the results of the commercial kits.

### 2.4. Statistical Analysis

The incidence of antibody loss was calculated per 1000 person-years from primary immunization to the latest follow-up.

To calculate and to compare the anti-HBs persistence between the poor, the moderate, and the good responders, life-table analysis using the Kaplan-Meier method and log-rank tests were used which take censoring into account and are therefore the preferred analysis to compare time to antibody loss. In the analysis, we defined time to antibody loss as an event and presented it as an estimated means.

Incidence density ratios adjusted for the variables of interest (titer after primary immunization, gender, age group, and type of vaccine) were estimated using Cox’s proportional hazards model to identify any factors associated with the loss of adequate anti-HBs titers. The analyses were carried out using the Statistical Package for the Social Sciences (IBM SPSS Statistics 26.0; SPSS Inc., Chicago, IL, USA).

## 3. Results

**Figure 1:**
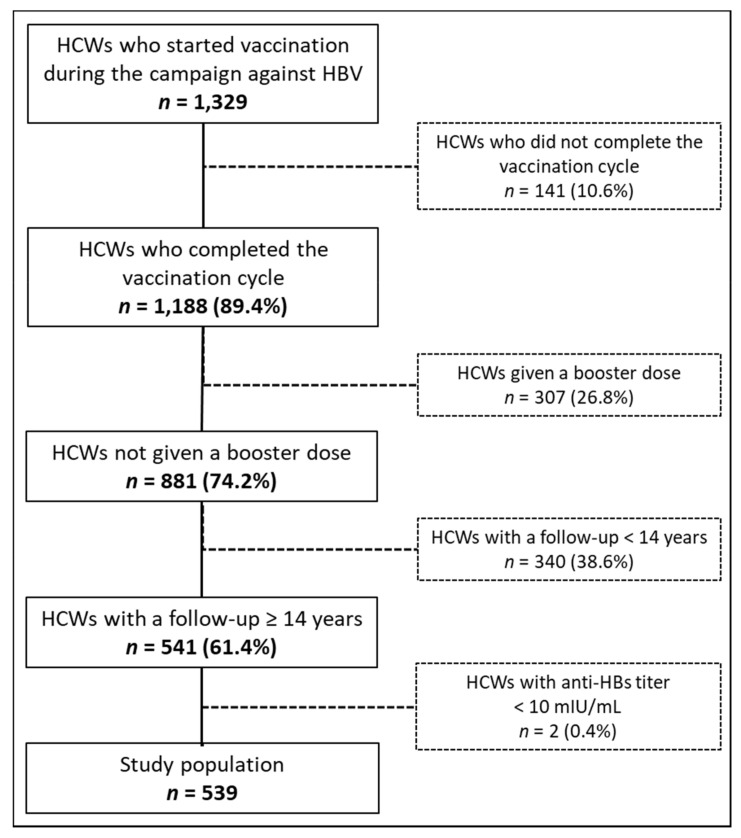
Flow diagram of the study group enrolment process.

From November 1983 to May 1993, based on our campaign against HBV, 1329 HCWs started the immunization cycle, and 141 of them were excluded from our analysis because they did not complete the primary HBV vaccination cycle. Of the 1188 HCWs that completed the immunization cycle, 307 (26.8%) received a booster dose, and 340 (38.6%) had a follow-up of fewer than 14 years, which left 541 HCWs eligible for our analysis. Overall response after primary immunization was 96.9% (539 HCWs). The flow-chart in Figure 1 summarizes the study group enrolment process.

Of the study population (539 subjects), 81.8% were female, and 59.4% were given the yeast-derived vaccine. Overall, the median age at the first vaccine dose against HBV was 21.0 years, the mean age was 21.6 ± 4.8 years, instead. According to gender, females were significantly younger than males, the mean ages were 21.1 ± 4.6 years and 23.8 ± 5.3 years, respectively (*p* < 0.001). Antibody titers after primary immunization identified 494 (91.7%) as good responders, 37 (6.9%) as moderate responders, and 8 (1.5%) as poor responders (who tested negative during the follow-up). The probability of antibody loss was significantly lower for the moderate and good responders than for the poor responders. To be specific, the incidence of anti-HBs loss was 52.1 per 1000 person-years in poor responders (used as the reference group in the logistic regression analysis), 11.3 per 1000 person-years for the moderate responders (adj RR (95% CI): 0.28 (0.10–0.80), *p* = 0.018), and 1.4 per 1000 person-years for the good responders (adj RR (95% CI): 0.03 (0.01–0.09), *p* < 0.001). No significant differences in the probability of antibody loss emerged in relation to gender (*p* = 0.986) or age group (*p* = 0.556). Regarding the type of vaccine, there was a trend towards greater anti-HBs loss in HCWs given the yeast-derived vaccines than in those given the plasma-derived vaccines without reaching a statistically significant difference (adj RR (95% CI): 2.1 (0.86–4.98), *p* = 0.107) (Table 1).

**Figure 2:**
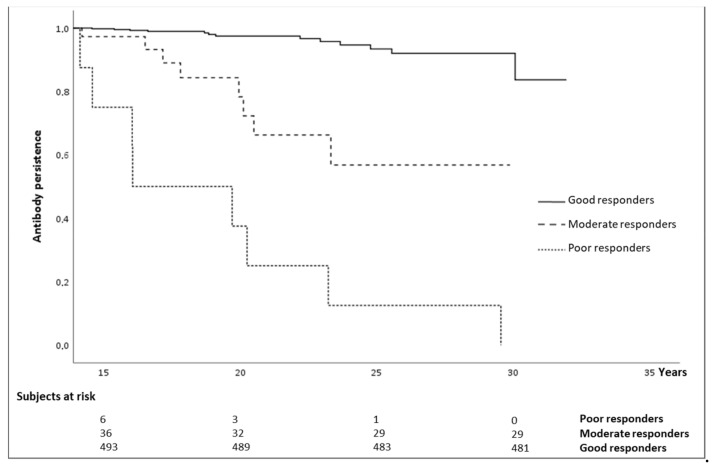
Anti-hepatitis B (HB) persistence by anti-HBs titers after primary vaccination.

The overall mean follow-up was 19.3 ± 4.5 years, with no significant differences between the groups of responders. The anti-HBs persistence up to 30 years after vaccination was 83.7% for good responders, and 56.8% for moderate responders, whereas anti-HBs persisted for up to 28 years in poor responders (12.5%). The mean persistence of anti-HBs differed significantly between the three groups, being: 19.2 years (95% CI: 15.6–22.8), 25.4 years (95% CI: 23.0–27.9), and 31.0 years (95% CI: 30.5–31.5) for the poor, moderate, and good responders, respectively (Kaplan-Mayer log-rank test *p* = 0.001) (Figure 2).

## 4. Discussion

To our knowledge, this is one of the few prospective studies to have been conducted on a selected group of healthy adults to assess the persistence of anti-HBs for up to 30 years after their primary immunization against HBV, with no subsequent booster dose.

Our results indicate that response to the primary vaccination cycle was very high (99.6%), in line with a response of 98.8% recorded by Floreani and colleagues [25]. We found that 91.7% of vaccinated HCWs had an anti-HBs titer higher than 1000 mIU/mL. For 6.9% of them, it was between 100–999 mIU/mL. For 1.5%, it was between 10–99 mIU/mL, and only 2 HCWs did not reach protective anti-HBs levels.

The persistence of anti-HBs during the follow-up correlated significantly with anti-HBs titer after primary vaccination: for good responders, the likelihood of experiencing loss of anti-HBs was only 3% of the corresponding risk for poor responders.

In our previous study [25] on 422 subjects, protective levels of anti-HBs persisted 10 years after primary immunization in >85% of cases, suggesting that a booster dose is unnecessary in healthy adults for 10 years. The present study shows that even 30 years after primary immunization, anti-HBs were still detectable in 84% of good responders and 56.8% of moderate responders, while 50% of poor responders had detectable anti-HBs 19 years after being immunized.

Other studies have also reported a significant relation between anti-HBs titer after primary vaccination and the persistence of anti-HBs over time. To be more specific, the findings of a retrospective cohort study conducted on HCWs from 2001 to 2012 showed that HCWs with higher initial anti-HBs titers after primary vaccination had significantly more persistent seropositivity. For short-term responders, a three-dose booster vaccination gave rise to a more durable anti-HBs positivity compared with a single-dose booster. For long-term responders, on the other hand, a single-dose booster sufficed to ensure a prolonged anti-HBs positivity [26]. Another study was performed to examine the persistence of immunity from 17 to 20 years after primary vaccination. This study involved more than 1300 healthy subjects. It found that, in the 25.5% of participants whose anti-HBs antibody titers were <10 mIU/mL, a challenge dose prompted a significant 93.1% increase in the number of individuals with an adequate anti-HBs positivity (≥10 mIU/mL) [27].

These findings support current recommendations issued by the Advisory Committee on Immunization Practices (ACIP) and the European Consensus Group. According to these recommendations, HCWs who have been vaccinated against HBV and have a documented immunity (anti-HBs concentrations of ≥10 mIU/mL) require no post-exposure prophylaxis, serological testing, or additional vaccination. This recommendation can be only valuable for “good” and “moderate” responders, however [14,24,28].

Recent studies reported an anamnestic response in more than 95% of subjects given a booster dose after revealing a non-protective anti-HBs titer (<10 mIU/mL) [29,30]. This was confirmed by research conducted on about 10,000 students in the Veneto Region in 2019, which showed that anti-HB levels higher than 2 and lower than 10 mIU/mL were predictive of a prompt response to a booster [31]. In contrast, another recent study suggested the need for a booster dose 10 years after primary vaccination, for healthcare professionals at least [32].

As concerns the type of vaccine used, our results confirm that subjects given the yeast-derived vaccine are twice as likely as those given the plasma-derived vaccine to have declining antibody levels [25], although this difference was not statistically significant in our sample. In a previous study of ours, individuals who were initially given plasma-derived vaccines were better seroprotected than those given yeast-derived vaccines. The former had significantly higher anti-HBs geometric mean titers (GMTs) during their follow-up, despite both groups reaching similar GMTs after primary immunization [25]. Studies on antibody kinetics have shown that, after an initial drop in titer during the first few months, the decline in antibody concentrations becomes much slower in subsequent years [33]. This gradual decline in anti-HBs titer also depends on the titer reached after the primary vaccination cycle [34]. Findings emerging from studies on infants have painted the same picture [35]. That said, there is no medical evidence to suggest that vaccinated healthy individuals lose their immunity simply because their anti-HBs levels drop below 10 mIU/mL. The GMT is only one of the variables to consider when judging an individual’s protection. Cellular immunity has a more important role in the anamnestic response, and the virus’s incubation period is longer than the time it takes to mount an anamnestic response. Finally, vaccination screening in HCWs is also useful to identify infected subjects with active HBV infection in order to reduce the transmission risk to patients. Indeed, according to the 2017 Clinical Practice Guidelines of the European Association for the Study of the Liver, HCWs performing exposure-prone procedures with serum HBV DNA >200 IU/mL may be treated with nucleos(t)ide analog [36].

The available evidence toward an effect of male gender on antibody loss is not robust: our findings are not able to suggest a role of the gender in the antibody loss at follow-up. While our findings are in line with most of the past literature, a study conducted by Wood and colleagues [37] showed a significant association between male gender and antibody loss, but this was significant only regarding Engerix-B recipients and not for Recombivax HB recipients. However, a more recent study by Yang and colleagues [38] showed an association between male gender and vaccine non-response. As suggested by the authors, part of the heterogeneity in the vaccine response may reside in the opposite effects of sex hormones on the epigenetic adjustment of genetic expression and in the gene structure on the X chromosome differing between XX females and XY male, but further research is needed in this specific field.

The main weakness of our study lies in the lack of data relating to HCWs’ specific features and behavior (alcohol consumption, smoking habits, obesity, sexual behavior, and so on), which are variables believed to affect the persistence of protective anti-HBs antibodies over time [39,40]. Another limitation of the study concerns the number of poor responders, but this is due to the generally good response to primary immunization. In addition, all participants involved in our study did not report any previous contact with the HBV prior to primary immunization and received a full course of anti-HBV vaccination as HCWs for the first time in their life at the time of recruitment (or starting of the internship) by their healthcare facility, in fact, the mean age of the studied population was of 20.0 years. This may pose some limitations to the extensibility of results to either the following cohorts of HCWs or the general population: in 1991 in Italy, indeed, a universal routine anti-HBV vaccination program was implemented, including both infants in the first year of age—with a schedule at three, five and eleven months, while vaccination at birth was recommended for only infants born to HBsAg positive mothers—and a catch-up strategy addressed to adolescents at the age of 12 for the first 12 years of application in order to cover, in only a dozen years, the whole Italian population aged 0–24 years. As shown by recent studies conducted by Chiara and Coppola, the rate of antibody loss correlates inversely with vaccination age while the level of antibody titer is directly related to the age of primary immunization [5,41].

On the other hand, our study can rely on a quite large cohort of more than 500 participants and, most of all, a considerably long follow-up time up to 30 years.

## 5. Conclusions

The present study demonstrated a good persistence of protective anti-HBs titers in HCWs at occupational risk of HBV for up to 30 years (even without a booster dose) if their starting titer after the primary vaccination cycle was greater than 100 mIU/mL. This result supports current guidelines, which do not recommend a booster dose for HCWs whose antibody titer after primary immunization was ≥10 mIU/mL.

## Figures and Tables

**Table 1:** Antibody loss at follow-up in responders to primary immunization.

Characteristics	HCWs		Anti-HBs Negative		Person-Years	Antibody Loss		
	*N*	(%)	*N*	(%)		Incidence Density/1000 Person-Years	Adjusted RR (CI 95%)	*p*
Overall	539	100.0	29	5.4	10414.6	2.8		
								
Titers after primary immunization								
10–99	8	1.5	8	100.0	153.6	52.1	-	-
100–999	37	6.9	8	21.6	707.6	11.3	0.28 (0.10–0.80)	0.018
≥1000	494	91.7	13	2.6	9553.3	1.4	0.03 (0.01–0.09)	0.000
								
Sex								
Females	441	81.8	22	5.0	8623.1	2.6	1.0 (0.40–2.50)	0.986
Male	98	18.2	7	7.1	1791.5	3.9	-	-
								
Age group								
≤20	267	49.5	9	3.4	5284.4	1.7	-	-
21+	272	50.5	20	7.4	5130.2	3.9	1.3 (0.50–3.20)	0.556
								
Type of vaccine								
Plasma-derived	219	40.6	8	3.7	4444.5	1.8	-	-
Yeast-derived	320	59.4	21	6.6	5970.1	3.5	2.1 (0.86–4.98)	0.107

## Data Availability

All relevant data are within the paper. Requests for additional information should be addressed to the corresponding author and data may be provided on reasonable request.
